# Wiring Up Along Electrodes for Biofilm Formation

**DOI:** 10.3389/fmicb.2021.726251

**Published:** 2021-08-30

**Authors:** María Belén Prados, Mariela Lescano, Natalia Porzionato, Gustavo Curutchet

**Affiliations:** ^1^Instituto de Energía y Desarrollo Sustentable, Centro Atómico Bariloche, Comisión Nacional de Energía Atómica, Buenos Aires, Argentina; ^2^Instituto de Investigaciones e Ingeniería Ambiental y Escuela de Ciencia y Tecnología, Universidad Nacional de San Martín, Buenos Aires, Argentina

**Keywords:** bacterial nanotubes, bioelectrochemical system, cable bacteria, geobacter, multispecies biofilm, shewanella, electrogenic bacteria

## Abstract

Millimeter-length cables of bacteria were discovered growing along a graphite-rod electrode serving as an anode of a microbial electrolysis cell (MEC). The MEC had been inoculated with a culture of Fe-reducing microorganisms enriched from a polluted river sediment (Reconquista river, Argentina) and was operated at laboratory controlled conditions for 18 days at an anode poised potential of 240 mV (vs. Ag/AgCl), followed by 23 days at 480 mV (vs. Ag/AgCl). Anode samples were collected for scanning electron microscopy, phylogenetic and electrochemical analyses. The cables were composed of a succession of bacteria covered by a membranous sheath and were distinct from the known “cable-bacteria” (family Desulfobulbaceae). Apparently, the formation of the cables began with the interaction of the cells via nanotubes mostly located at the cell poles. The cables seemed to be further widened by the fusion between them. 16S rRNA gene sequence analysis confirmed the presence of a microbial community composed of six genera, including *Shewanella*, a well-characterized electrogenic bacteria. The formation of the cables might be a way of colonizing a polarized surface, as determined by the observation of electrodes extracted at different times of MEC operation. Since the cables of bacteria were distinct from any previously described, the results suggest that bacteria capable of forming cables are more diverse in nature than already thought. This diversity might render different electrical properties that could be exploited for various applications.

## 1. Introduction

The current depletion of the world's non-renewable resources and the need to reduce anthropogenic effects on the environment have encouraged the development of new production systems and waste treatment plants. In this way, bioelectrochemical systems (BES) represent a versatile technological platform capable of converting organic pollutants dissolved in wastewaters into a renewable energy source. BES are mainly studied for the production of electricity and hydrogen but also different valuable products can be produced (Angenent et al., 2004; Nevin et al., 2011; Logan and Rabaey, 2012; Wang et al., 2015). Most recently, the versatility of BES has turned them into a novel approach for the immobilization and recovery of heavy metals from wastewaters (Wang and Ren, 2014; Puyol et al., 2017).

BES are designed to exploit the ability of certain microorganisms to transfer electrons to solid extra-cellular acceptors during cellular respiration, through a process called *extra-cellular electron transfer* (EET), which has been extensively reviewed (Lovley, 2006, 2012; Kracke et al., 2015). These microorganisms are known as electrogenic, electroactive (EAB), or anode-respiring bacteria (ARB) since they can respire and conserve energy with an electrode serving as the sole electron acceptor (Bond et al., 2002; Bond and Lovley, 2003). Interestingly, some of these microorganisms can also gain electrons from an electrode and reduce soluble electron acceptors. In natural environments, most of the currently described electrogenic microorganisms are capable of coupling the complete oxidation of an organic compound to CO_2_ to the dissimilatory reduction of insoluble Fe(III) and Mn(IV) oxides (Lovley et al., 2004). This anaerobic metabolism is of particular geochemical relevance in submerged soils, aquatic sediments, and subsurface environments since it is the main mechanism for organic matter oxidation and can influence the fate of diverse trace metals. In addition, some electrogenic bacteria can also support growth by the reduction of soluble metals, such as U(VI) and Cr(VI). This potential has opened the possibility of developing BES for bioremediation and metal recovery (Gregory and Lovley, 2005; Tandukar et al., 2009). Microbiological reduced metals, like U(IV) or Cr(III), may settle on the electrode or precipitate at the bottom of the reactor and can, thus, be recovered.

Consequently, BES could overcome the challenge that poses heterogeneous wastewaters or contaminated environments which include organic waste from domestic origin and recalcitrant pollutants from industrial discharges. A clear example of such an environment is represented by the Reconquista river in Argentina. This river is located in the north of Buenos Aires province and it crosses 18 densely populated districts (more than four million people) along its 82 km route. The pollution of the Reconquista is mainly related to the discharge of domestic and industrial wastewaters that are poured almost untreated into the river (Castañé et al., 2006). The high oxygen demand determined in this river, generated by the oxidation of the organic matter, causes an anoxic environment which in turn favors bio-catalyzed sulfide formation. Under the low redox potential of the rivers water, heavy metals are precipitated or adsorbed on different mineral components of the sediment, turning it into a heavy metal reservoir (Porzionato et al., 2013). A thorough characterization of the rivers water and sediment was previously performed by our group (Tufo et al., 2018, 2021). The Reconquista river has high levels of chromium, among other diverse pollutants, and being the second most polluted river in Argentina it represents an acute environmental problem (Porzionato et al., 2016).

Therefore, with the aim of establishing a laboratory-scale prototype of a BES to recover heavy metals from the Reconquista river, we developed a strategy to select autochthonous electrogenic microorganisms from the sediment. This strategy allowed us to isolate an electrogenic community with an astonishing electrode colonization strategy, capable of forming bundles of cables of bacteria.

## 2. Materials and Methods

### 2.1. Sampling Site and Description

Sampling site is located at José León Suarez, Buenos Aires province, Argentina (geographic coordinates: 34°31'19.4”S, 58°35'28.0”W). The samples were taken from the José León Suarez channel, which carries pollutants due to the sewage and industrial upstream discharges. Sampling was performed as previously described (Porzionato et al., 2016). Briefly, superficial composite samples of the sediment were taken (0–20 cm depth), placed in plastic containers and stored at 4°C. Samples were bottled with their initial moisture content and kept saturated to maintain the anoxic conditions during storage. Before experiments, samples were manually homogenized and sparged with ultra high purity grade N_2_:CO_2_ (80:20).

### 2.2. Enrichment of Fe-Reducing Bacteria

A river sediment sample (16 mg) was inoculated into 50 mL of enrichment culture medium, the vial was sealed with a butyl rubber stopper in order to guarantee anaerobiosis and cultured at 30°C (“enrichment” vial). For control purposes, a similar vial was inoculated with a previously autoclaved sediment sample (“control” vial). The enrichment medium contained (per liter): 0.1 g of KCl, 1.5 g of NH_4_Cl, 0.6 g of NaH_2_PO_4_, 2.5 g of NaHCO_3_, 10 mL of vitamin mix and 10 mL of trace mineral mix (Atlas, 2010). Sodium acetate (30 mM) served as the electron donor and as the carbon source and Fe(III)-citrate (56 mM) as the electron acceptor. All reagents were from Sigma-Aldrich (analytical grade). The medium was adjusted to pH 6.8, sterilized and flushed with ultra high purity grade N_2_:CO_2_ (80:20) to remove oxygen. Cell growth and Fe (III) reduction were monitored spectrophotometrically. Gas production was analyzed by Fourier Transformed Infrared Spectroscopy (FTIR). In order to select the electrogenic microorganisms, once Fe(III) was mostly reduced, steady-state cells were anaerobically transferred to an electrochemical cell (10% inoculum).

### 2.3. Selection of Electrogenic Bacteria

We employed microbial electrolysis cells (MECs) to carry out the electrogenic bacteria selection and the experiment controls. The MECs consisted of sterile anaerobic glass single-chamber cells (200 mL) with three electrodes: a solid graphite rod (constructed from bars of 0.4 cm in diameter and 10 cm in length) served as the working electrode, a coiled platinum wire (0.5 mm diameter) was used as counter electrode and a Ag/AgCl electrode was used as reference electrode. The graphite rods were polished with sandpaper (P1000 grit), thoroughly rinsed with distilled water and sonicated in a bath (1 min, 3 times) before use. Three cells were prepared and filled with a slightly modified enrichment culture medium. In order to favor cell attachment and growth in the working electrode no electron acceptor was added. The cells were continuously flushed with N_2_:CO_2_ (80:20) and operated in an incubator at constant temperature (30°C) and stirring. Two MECs were inoculated with 10% of the “enrichment” culture (MEC-a and MEC-b) and the third MEC was inoculated with 10% of the “control” culture (MEC-c). The working electrodes of MEC-a and MEC-c were poised at +240 mV vs. Ag/AgCl (PGSTAT101 potentiostat, Metrohm AG, Switzerland) and the current intensity was recorded throughout the experiment. The circuit of MEC-b was left open.

### 2.4. Cyclic Voltammetry (CV)

CVs (PGSTAT101 potentiostat, Metrohm AG) were performed at the following cycling conditions: +240 to −550 mV, starting at 0 mV at 10 mV/s, 10 cycles. CVs were performed without stirring and without gas flushing. CVs were run before inoculation and at different times of MEC operation.

### 2.5. Fourier Transformed Infrared Spectroscopy (FTIR)

Gas samples were collected during Fe-reducing bacteria enrichment and electrogenic bacteria selection every 48 h in a degassed quartz cell with KBr windows. FTIR measurements were carried out in a Perkin Elmer Spectrum 400 spectrophotometer (MCT detector).

### 2.6. Scanning Electron Microscopy (SEM)

Electrodes were fixed in glutaraldehyde 2.5% over-night, dehydrated by immersion in a series of ethanol solutions (40, 60, 80, and 100% ethanol in ultra-pure water), air dried and sputtered with gold for the observation by SEM. Samples were observed in a FEI-NOVA Nano SEM 230 at an accelerating voltage of 5 kV. Energy-dispersive X-ray spectroscopy (EDS) was performed in some areas of the samples at 20 kV.

### 2.7. Phylogenetic Analysis

Bacterial diversity was analyzed by 16S-based tag-encoded FLX amplicon pyrosequencing, bTEFAP (Dowd et al., 2008), at MR DNA (www.mrdnalab.com, Shallowater, TX, USA). For this purpose, the microorganisms attached to the working electrode were grown at 30°C in anaerobiosis in the enrichment culture medium slightly modified (the Fe-citrate was replaced by Na-fumarate). When the culture reached the exponential growth phase, the genomic DNA was extracted with the GeneJET Genomic DNA purification kit (Thermo Fischer Scientific, Lithuania), following the manufactures guidelines. Then, the 16S rRNA gene V4 variable region PCR primers 515/806 with bar-code on the forward primer were used in a 28 cycle PCR (5 cycle used on PCR products) using the HotStarTaq Plus Master Mix Kit (Qiagen, USA) under the following conditions: 94°C for 3 min, followed by 28 cycles of 94°C for 30 s, 53°C for 40 s, and 72°C for 1 min, and then a final elongation step at 72°C for 5 min was performed. After amplification, PCR products were checked in 2% agarose gel. Multiple samples were pooled together (e.g., 100 samples) in equal proportions based on their molecular weight and DNA concentrations. Pooled samples were purified using calibrated Ampure XP beads. Then the pooled and purified PCR product were used to prepare illumina DNA library. Sequence data were processed using MR DNA analysis pipeline (MR DNA, Shallowater, TX, USA). Briefly, sequences were joined, depleted of bar-codes, then sequences <150 bp and ambiguous base calls were removed. Sequences were denoised, operational taxonomic units (OTUs) generated and chimeras removed. OTUs were defined by clustering at 3% divergence (97% similarity). Final OTUs were taxonomically classified using BLASTn against a curated database derived from RDPII and NCBI (http://rdp.cme.msu.edu, www.ncbi.nlm.nih.gov).

## 3. Results

### 3.1. Electrogenic Microorganisms Were Selected From a Polluted River Sediment

Based on the metabolic capacity of most of the currently described electrogenic microorganisms to couple the oxidation of an organic compound using extracellular iron oxides as electron acceptors, the selection strategy was divided into two stages: (1°) enrichment of Fe-reducing microorganisms from sediments, using a selective culture medium with Na-acetate as electron donor/carbon source and Fe-citrate as electron acceptor; (2°) selection of electrogenic microorganisms from the enrichment of Fe-reducing microorganisms, using a microbial electrolysis cell (MEC).

**Figure 1:**
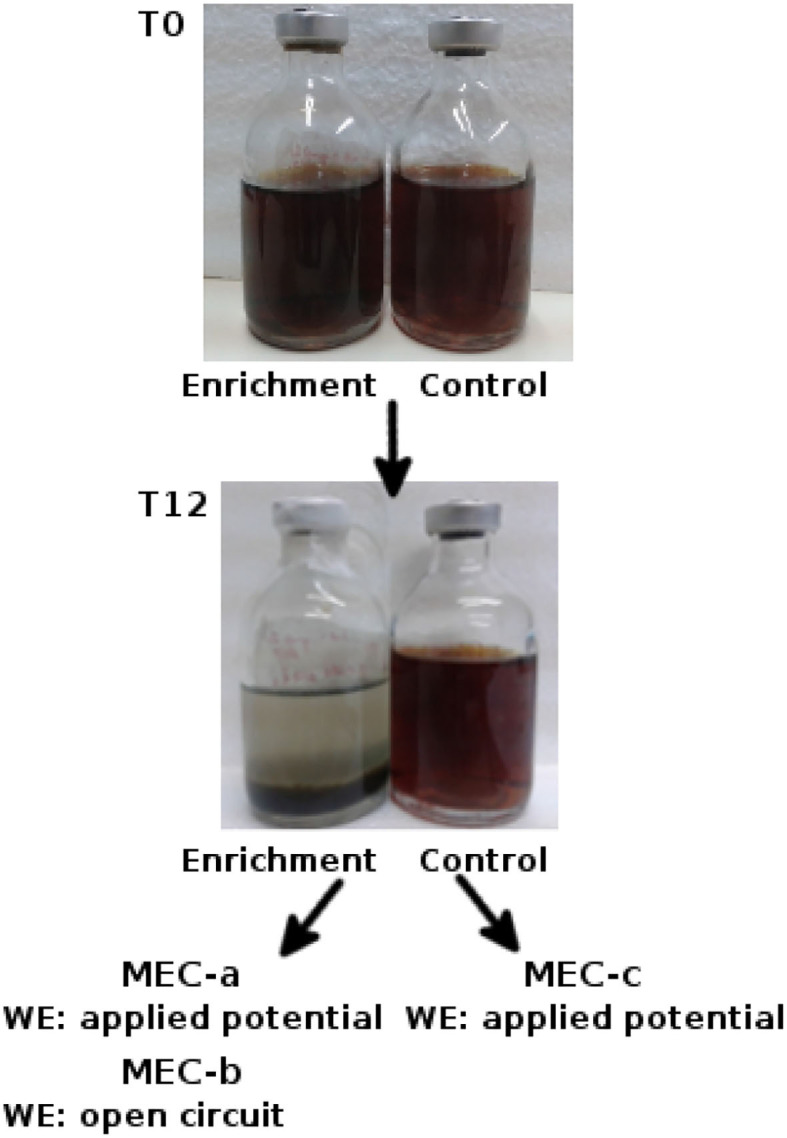
Selection of electrogenic microorganisms from a river sediment. T0 = anaerobic vials inoculated with the river sediment, “enrichment,” or with the sterilized river sediment, “control.” T12 = 12 days after inoculation. Two microbial electrolysis cells (MEC-a and MEC-b) were inoculated with the “enrichment” at T12 and one with the “control” (MEC-c). The working electrode (WE) of both MEC-a and MEC-c was initially poised at +240 mV vs. Ag/AgCl, whereas MEC-b was left at open circuit. The electrogenic microorganisms are expected to be recovered from MEC-a. MEC-b and MEC-c are control experiments: MEC-b was designed in order to analyze the possibility that there are microorganisms capable of forming a non-electrogenic biofilm over the electrode and MEC-c was carried out in order to understand electrochemical signals which may not arise from active growing bacteria.

Three MECs were prepared: MEC-a, MEC-b, and MEC-c. The MECs were inoculated when Fe^3+^ reduction and microbial growth were observed in the “enrichment” vial, which occurred after 12 days (T12) of culture (Figure 1). This was evidenced by a change in the color of the medium (from dark brown to colorless) and an increased turbidity, respectively. In the “control” vial, which was inoculated with the sterilized sediment, neither Fe^3+^ reduction nor microbial growth were observed during this time. MEC-a and MEC-b were inoculated with the “enrichment” culture and MEC-c with the “control” culture at T12.

**Figure 2:**
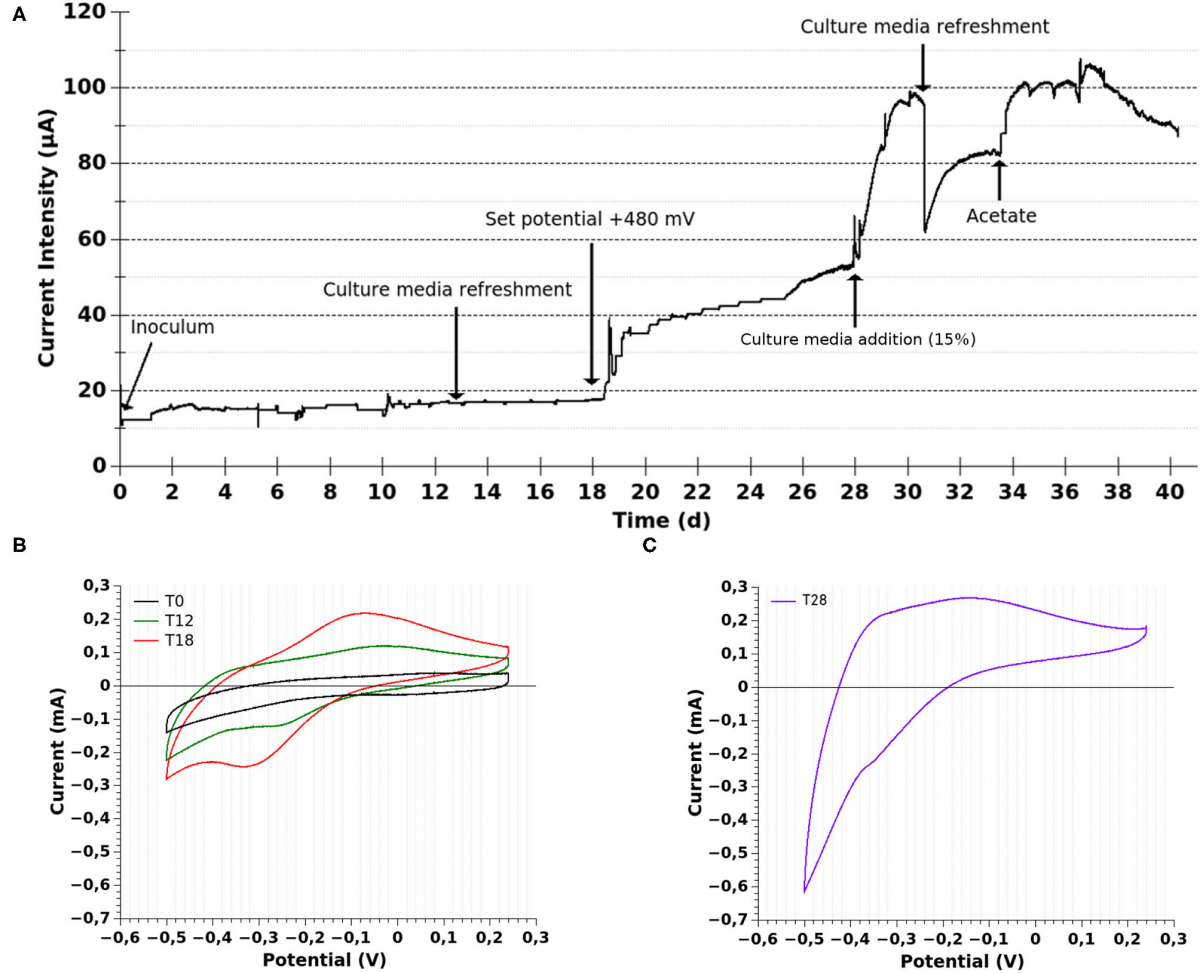
Electrochemical characterization of MEC-a. **(A)** Chronoamperometry. The current evolution of the MEC employed for the selection of electrogenic microorganisms is shown. The MEC was operated in batch mode at constant temperature (30°C) during the whole experiment. The MEC was inoculated with the “enrichment” culture (Fe-reducing microorganisms, 10%). The working electrode was initially poised at 240 mV vs. Ag/AgCl and changed to 480 mV vs. Ag/AgCl at T18 (after performing CV). **(B)** Cyclic voltammetry (CV) was performed at 10 mV/s before inoculation (T0), at 12 (T12), and 18 (T18) days of MEC operation. **(C)** CV at 5 mV/s at 28 days (T28) of MEC operation.

The current intensity of MEC-a showed a slight increasing tendency from T1, 24 h after inoculation (Figure 2A). CV analysis at day 6 revealed a reversible redox process consistent with electrogenic activity. These peaks were also seen at days 12 and 18 (Figure 2B). The midpoint potential of the redox process was centered at ~−195 mV vs. Ag/AgCl, which is in the potential window of the outer-membrane Mtr/Omc cytochromes of *Shewanella* (Carmona-Martinez et al., 2011). The differences in peak high and position between the different CVs are in agreement with a growing biofilm. Additionally, at day 8, viable cells (capable of growing in LB, acetate:fumarate and acetate:Fe-citrate) were recovered from the MEC medium (data not shown). Therefore, we decided to refresh the whole culture medium at day 13. This refreshment did not produce any significant change in the current production rate (Figure 2A), suggesting that the possible electrogenic bacteria present in the MEC were already attached and were growing at the electrode surface employing at least a direct electron transfer mechanism, as observed by CV.

Thereupon, in order to study the conditions which could promote a higher current production, at day 18 (after performing the mentioned CV) we set the WE potential at a more positive value, closer to Fe-oxides (from +240 to +480 mV vs. Ag/AgCl; Figure 2A). Afterwards, the current significantly increased. Then, at day 28 (T28), when a plateau in the current intensity was being reached, fresh culture medium was added (15% of MEC volumen; leading to at least 4 mM of Na-acetate). This modification prompted an abrupt increase in the current intensity followed by a higher current production rate, suggesting that the electron donor was reaching a limiting concentration and also that the fresh medium favored the electron transfer. Interestingly, CV performed at T28 showed a catalytic wave with an inflection point centered at −400 mV (Figure 2C), consistent with the presence of a denser biofilm that was oxidizing acetate at the electrode surface. Additionally, a redox process centered at ≈−350 mV could be appreciated. This result might indicate the presence of another electron transfer mechanisms, which was not previously observed. On the one hand, some electrogenic bacteria are capable of producing different membrane-bound proteins that could account for the observed redox process (Sharma et al., 2016). On the other hand, soluble electron shuttles, such as flavins, could also be involved. This would be consistent with previous reports of *Shewanella putrefaciens* grown at a poised graphite electrode (+400 mV vs. Ag/AgCl) with the addition of 1 μM of riboflavin (Carmona-Martínez et al., 2013). It is important to note that the fresh culture medium included riboflavin at 0.1 μM, which was not detected in the CV at T0. Therefore, if the observed signal at T28 was related to soluble mediators, they were synthesized by the bacteria. Nonetheless, the riboflavin added in the fresh medium could have favored the fastest current production rate of the growing biofilm.

The difference between the CVs of the biofilm grown at +240 vs. +480 mV could suggest that the biofilm adapted to the higher electrode potential by modifying the electron transfer mechanisms. This would be in agreement with previous evidence showing that electrogenic bacteria can exploit current-generating mechanisms that are suited for a given electrode potential (Kitayama et al., 2017). However, considering that we performed the CVs over a growing biofilm we can not discard the possibility that in our case, the differences observed are not related to a change or addition of an electron transfer mechanism in response to a change in the set potential but to an increased and detectable contribution of an already present mediator.

The maximum current intensity was reached at day 31 and, at this time, a reddish color could be observed on the surface of the working electrode. When the current stabilized, the culture medium was completely replaced by fresh medium, producing a sudden and abrupt decrease in the current intensity (≈37.5%), which took 3 days to reestablish. This result could suggest the presence of soluble electron shuttles, synthesized by bacteria, which were contributing to current generation or it could only be due to a disturbance of the biofilm activity produced during the medium change. If flavins were involved, the removal of the whole culture medium would have probably not removed the total flavin content, since flavins can bind to carbon electrodes and cell material (Marsili et al., 2008). Considering that electron mediators are recycled by the cells but the electron donor may reach limiting concentrations with the cell operated in batch mode, when the current intensity reached a plateau (T34), we added Na-acetate, which induced a higher current intensity.

On the other hand, the current intensity of MEC-c remained stable and close to zero during the whole experiment. No significant changes were observed after modifying the operational parameters as described for MEC-a neither in the chronoamperometry nor in cyclic voltammetry (data not shown).

The pH of the three MECs culture medium was stable (around 7) during the entire operation.

**Figure 3:**
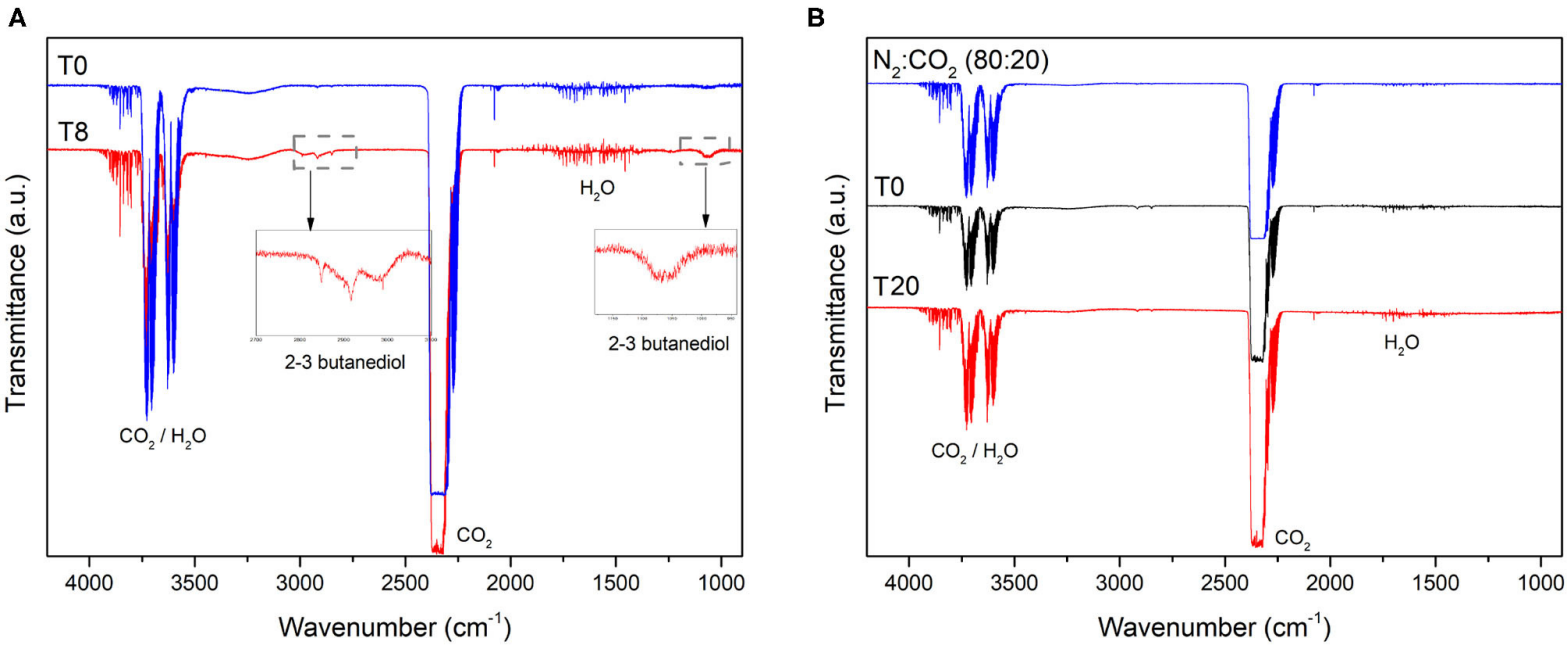
Gas composition of the **(A)** enrichment vial and **(B)** MEC-a head-space determined by Fourier Transformed Infrared Spectroscopy (FTIR). The enrichment vial was sparged with N_2_:CO_2_ (80:20) at T0 and then inoculated with the river sediment. A gas sample was collected from the head-space 8 days after inoculation (T8). MEC-a was inoculated with the “enrichment” culture after 12 days of incubation of the vial as described in section 2. The MEC was kept in anaerobiosis by sparging with N_2_:CO_2_ (80:20, blue line in **B**). The working electrode of MEC-a was poised at +240 mV vs. Ag/AgCl at T0. Gas samples were collected from the MEC head-space every 48 h. Considering that no differences were observed from day 0 (T0) of MEC operation, only T0 and T20 are shown for clarity purposes. The spectrum of MEC-b and MEC-c were similar to that of MEC-a.

As expected, since CO_2_ was employed (along N_2_) for creating the anaerobic atmosphere of vials and MECs, it was detected by FTIR in the vials and all the MECs before inoculation (T0) and during the course of the experiment (Figure 3). However, CO_2_ might also arise as a consequence of complete oxidation of organic compounds by growing microorganisms. Additionally, 2,3-butanediol was detected in the enrichment vial (Figure 3A). This might be the product of citrate fermentation by lactic bacteria (Hugenholtz, 1993). This compound was not detected during the bioelectrochemical cells operation, since citrate was not provided in the MECs medium and probably the bacteria which consume it were no longer viable (Figure 3B). No gases other than CO_2_ were detected in any of the MECs during the whole experiment.

Therefore, these results show that an electrogenic community was selected and recovered from the sediments of the heavily polluted José León Suarez channel of the Reconquista river.

After 41 days of operation, the WEs were removed and sectioned in two parts: one half was prepared for electron microscopy and the other half was sub-cultured in complete medium with acetate:fumarate to recover and characterize the microorganisms that were able to grow over the electrode.

### 3.2. Cables of Bacteria Ran Along the Electrode Surface

**Figure 4:**
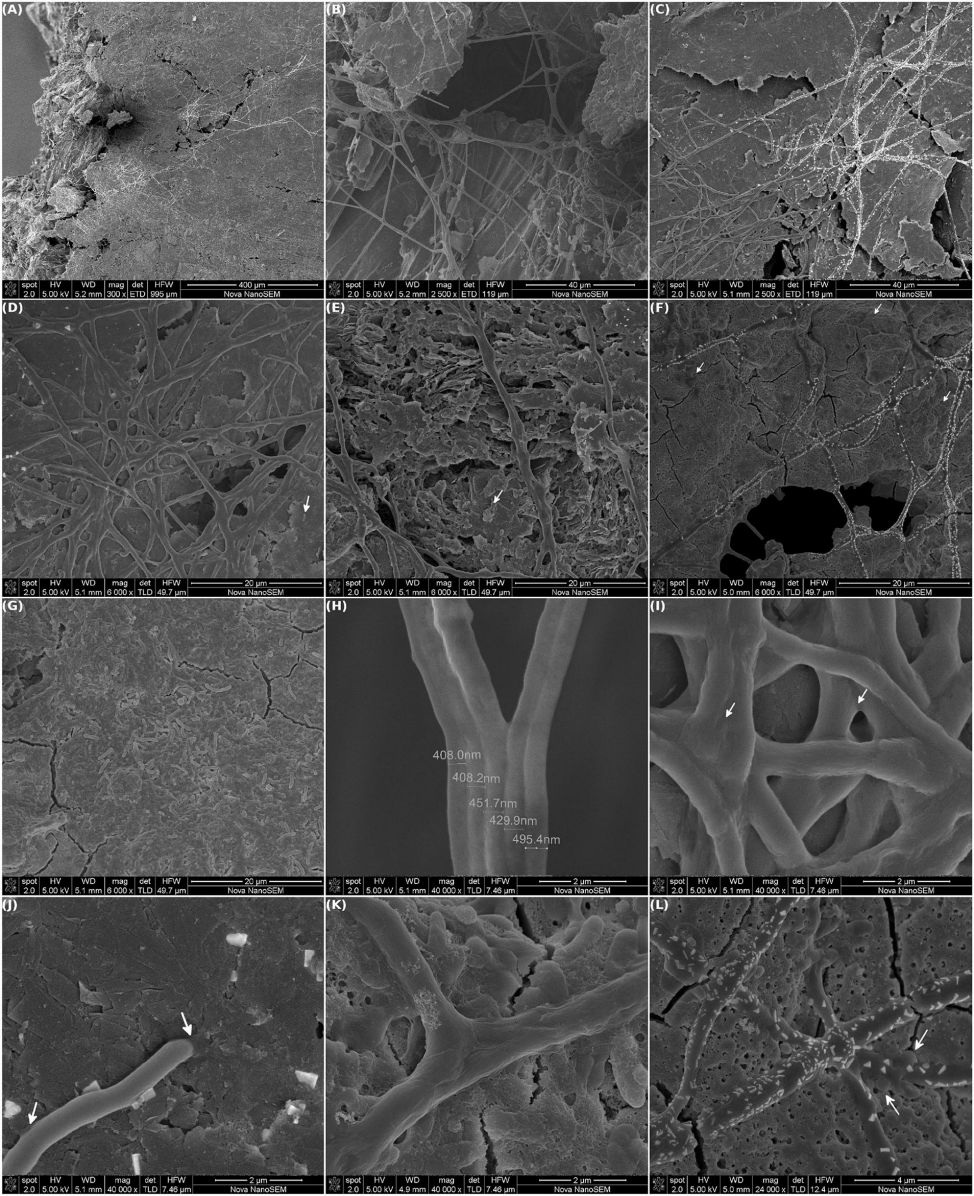
Scanning electron microscopy of MEC-a working electrode (WE). The MEC was inoculated with the Fe-reducing microorganisms enriched from the river sediment and the WE (graphite) was initially polarized at +240 mV vs. Ag/AgCl. The MEC was operated at 30°C in anaerobiosis, other operational parameters were modified as specified in Figure 2. At the end of the experiment (41 days) the WE was removed and observed by SEM: **(A–C)** Intertwined millimeter long cables of bacteria running along the WE surface. **(D–G)** Differences in the biofilm structure were observed over the electrode: zones of high density of cables and low density of isolated cells (**D**, white arrow), zones with low density of cables and higher density of aggregated cells **(E)** and biofilm matrix (**F**, white arrows) and **(G)** zones with no cables. **(H)** Single cables width. **(I)** Fusion of several cables. **(J)** Points of intimate contact with the electrode surface, white arrows. **(K,L)** The cables extended in many directions and have blebs (white arrows).

Eye-catching millimeter long cables of bacteria were discovered running along the surface of the working electrode from MEC-a (Figure 4). The biofilm was mostly composed of intertwined cables that could even form straight bridges over voids in the graphite (Figures 4A–F). The chained bacteria were covered by a continuous and smooth sheath. The appearance of this sheath, among other features, is distinctive from what is observed in the already described “cable-bacteria” (Desulfobulbaceae), which shows continuous longitudinal ridges over cell-cell junctions (Trojan et al., 2016).

As can be appreciated in Figures 4A,C,F,L, some areas of the cables showed a specific deposition of small crystals of different shapes. These crystals included different elements, mainly Fe, P, Na, and K, as determined by energy-dispersive X-ray spectroscopy (EDS, Supplementary Figure 1A).

Interestingly, differentially colonized electrode areas could be distinguished. In areas with a high density of cables, which co-occurred with areas of plain graphite sheets, only a few isolated bacteria were observed attached to the electrode (Figure 4D, white arrow). In contrast, in electrode areas with a lower cable density and sheared graphite more aggregated cells were found (Figure 4E, white arrow). There were also low density cable areas where the cables extended over a dense biofilm matrix. This matrix was composed mainly of EPS and isolated microcolonies (Figure 4F, white arrows). Additionally, areas with no cables and a denser biofilm with morphological diverse cells were observed (Figure 4G). It is known that growth-phase affects the size, shape, and cell wall thickness of bacteria (Cushnie et al., 2016). However, as confirmed by 16S phylogenetic analysis (see section 3.4), these results indicate that a community instead of a single specie of microorganism was selected. Moreover, they suggest that the different bacterial species were initially segregated over the electrode surface.

Single cables were composed of a cell width of ~450 nm and many bacteria in length (Figure 4H). The cables seemed to be further widened by fusing each others sheath and, thus, forming bundles of filaments (Figure 4I). The extremes of the cables were rounded and appeared to be, among other points, a site of intimate contact with the electrode surface (Figure 4J, white arrows). Remarkably, not only single cables extended in many directions, but they could also branch (Figure 4K). Intriguingly, “cable hubs” (resembling city highways) were observed along the electrode (Figures 4A–F,L, higher magnification). Since we do not know whether there was a cable growing polarity, we can not discern whether the cables converge or diverge from the hubs. In addition, some protrusions, that look like outer membrane blebbing (sometimes referred also as “blistering” and “bubbling”), were identified arising from the cables (Figure 4L, white arrows).

**Figure 5:**
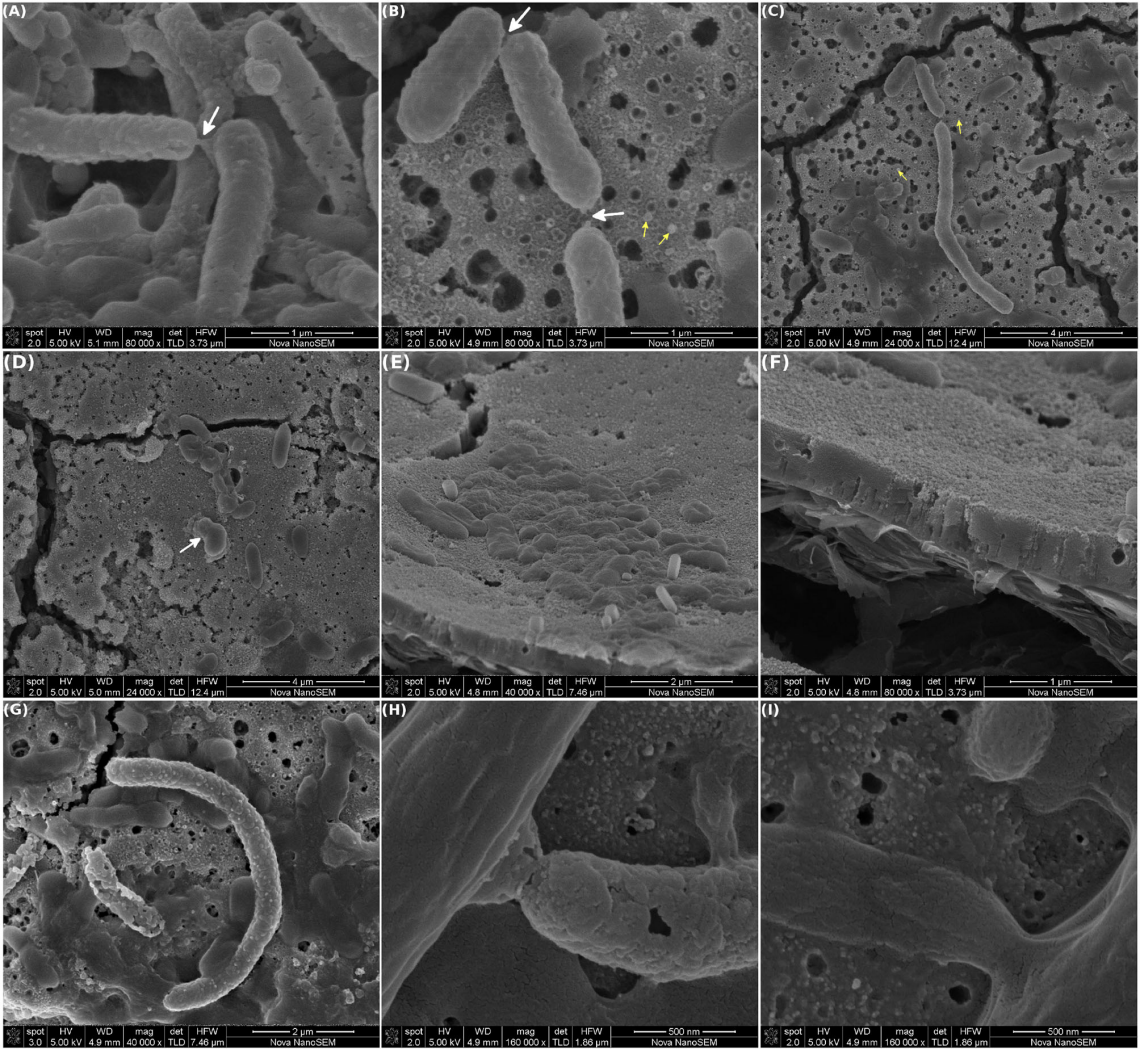
Scanning electron microscopy of MEC-a working electrode (WE). The MEC was inoculated with the Fe-reducing microorganisms enriched from the river sediment and the WE (graphite) was initially polarized at +240 mV vs. Ag/AgCl. The MEC was operated at 30°C in anaerobiosis, other operational parameters were modified as specified in Figure 2. At the end of the experiment (41 days) the WE was removed and observed by SEM. **(A–C)** Cells connected via nanotubes, white arrows (in **B,C** the tubes broke down during image acquisition). Yellow arrows point out at outer membrane vesicles. **(D)** Blebbing in an individual cell. **(E)** Cells over and embedded in an EPS deposit. **(F)** Close-up on a section of the deposit, showing the granular texture. **(G,H)** Biomineralized cells and **(I)** non-mineralized cables.

Interestingly, nanotubes connecting individual cells were observed. These tubes were mainly located at the cell poles (Figures 5A,B, white arrows) and were seen in short length cables or filaments (≈7–8 cells; Figure 5C). In addition, nanotubes were present in cells that showed a high degree of outer membrane vesiculation, which are distinguished by the small and rough blebs in the cell surface. Small particles (≈100 nm), compatible with free outer membrane vesicles (OMV) produced by Gram negative bacteria, were observed in the vicinity of these cells (Figures 5B,C, yellow arrows). Additionally, individual cells with single and bigger outer membrane protrusions were also observed (Figure 5D, white arrow).

Some cells seemed to be over and others embedded in a deposit that showed a granular texture (Figures 5E,F). This deposit might be composed of EPS and was rich in Fe, P, Na, and K as determined by EDS (Supplementary Figure 1B). Interestingly, mineralization of the cell wall was observed in isolated cells, as well as, in some incipient cables (Figures 5G,H). Figure 5I shows a non-mineralized cable at the same magnification of Figure 5H for comparison purposes. Mineralization trapped the cells in a shell of insoluble minerals, which ended the life of the microbial cell, as described previously (Orange et al., 2013; Jroundi et al., 2017). Noteworthy, biomineralization is now considered a process that plays a significant role in the development of fine-grain minerals in soils and sediments and is currently under intensive research (Cuadros, 2017).

On the other hand, in the WEs of both MEC-b and MEC-c neither bacterial growth nor significant bacteria attachment were observed (Supplementary Figure 2).

Therefore, these results show that at least one of the members of the electrogenic community selected from the Reconquista river sediment present an amazing cable-type biofilm development, which might be advantageous in poised surfaces.

### 3.3. The Bacterial Cables Dispersed Once the Electric Circuit Was Opened and a Soluble Electron Acceptor Was Provided

**Figure 6:**
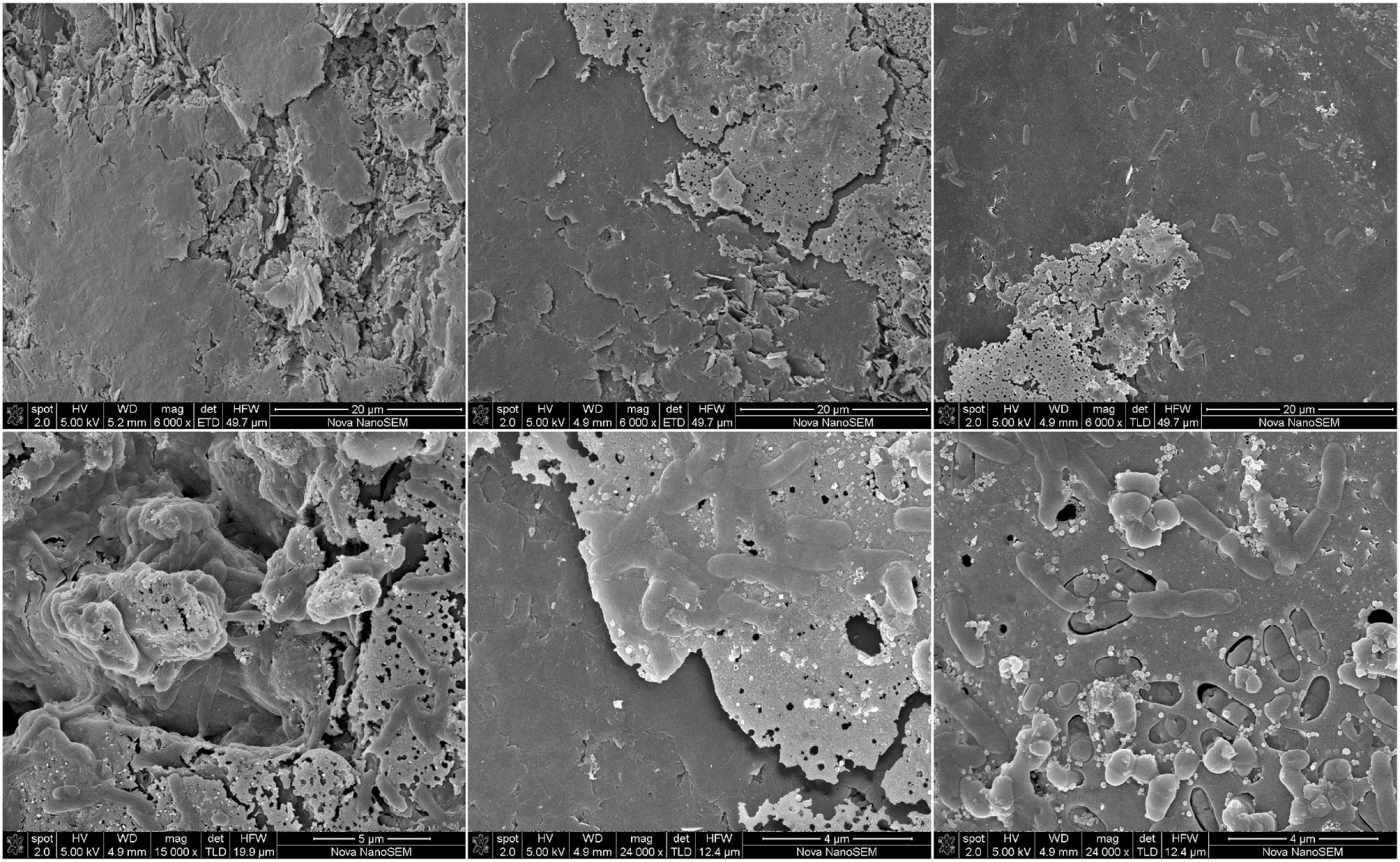
Scanning electron microscopy of MEC-a working electrode (WE) after being removed from the cell and cultured in acetate:fumarate in anaerobiosis during 21 days. Different areas of the graphite are shown at low **(Top)** and high magnification **(Bottom)**. No cables of bacteria were found, and the cells were mostly dispersed over the surface. Imprints of removed cells can also be distinguished.

As mentioned before, after 41 days of operation the WEs were removed and sectioned in two parts. One piece of the electrode was observed by SEM and the results were discussed above. The other piece of the electrode was placed in an anaerobic vial with culture medium, which included Na-acetate as electron donor and carbon source and Na-fumarate as electron acceptor. This vial was cultured for 21 days at 30°C. An increasing turbidity, consistent with microbial growth, was observed during this time. Then, the graphite bar was recovered for SEM inspection (Figure 6) and the microorganisms present in the culture medium were separated in three fractions: one was preserved in liquid nitrogen for further characterization, the second was subjected to 16S phylogenetic analysis (discussed in the next section of the manuscript) and the third one was inoculated in another electrochemical cell (presented in sections 3.4 and 3.5).

In Figure 6, different areas of the remaining WE from MEC-a are shown at different magnifications. No cables neither remainders of them were found, only individual cells or groups of at most three cells with the cells boundaries clearly delimited were observed. As well, as can be easily appreciated, most of the dense biofilm matrix present in other parts of the WE was also dissolved. Only few dispersed cells were found adhered to the graphite surface. Interestingly, a high concentration of vesicles was observed. As previously mentioned, these vesicles are similar to OMV produced by Gram negative bacteria and, in this case, they were present mostly as free vesicles.

The biofilm detachment or dispersal is regarded as a strategy of bacterial cells to leave biofilms when the environmental conditions within it are no longer favorable (Muhammad et al., 2020). In addition, vesiculation was previously described as an independent and flexible process for stress management (Mcbroom and Kuehn, 2007) and is regarded as a survival advantage in mixed bacterial populations. Since OMV may have proteolytic activity, they can help in nutrient acquisition (Ellis and Kuehn, 2010).

Thus, these results confirm that the cable-type biofilm was dependent, at least, on the electrode polarization.

### 3.4. The Selected Community Include Anode Respiring Bacteria

Bacterial diversity of the selected community was analyzed by 16S pyrosequencing, where the operational taxonomic units (OTUs) thus generated were compared to reference databases to infer taxonomy. Bacteria belonging to three classes were identified: Clostridia, γ-proteobacteria, and Bacteroidia (Table 1). Considering that the study was performed over the community growing in liquid medium with acetate:fumarate, the relative proportion of each specie growing over the electrode might not be exactly the same as the one reported here. In this condition, the community was mainly dominated by Clostridia and γ-proteobacteria. Among these, the 98.54% of the counts corresponded to four genera where electrogenic members were already described: *Clostridium, Aeromonas, Shewanella*, and *Enterobacter* (Kim et al., 1999; Park et al., 2001; Pham et al., 2003; Feng et al., 2014). In particular, the most abundant OTU (99.83%) for the genus *Shewanella* had a 99.27% identity with *S. putrefaciens*. This was the only OTU that could be classified to a unique specie. The OTUs for the other genera that could have been classified to the species level (identity percentage >97%) matched with several species of the specific genus.

**Table 1:** Phylogenetic affiliation of the operational taxonomic units (OTU) detected by 16S sequencing.

**% OTU**	**Class**	**Order**	**Family**	**Genus**	**# OTU**
56.74	Clostridia	Clostridiales	*Clostridiaceae*	*Clostridium*	2
0.02	Clostridia	Clostridiales	*Lachnospiraceae*	*Roseburia*	1
31.10	γ-proteobacteria	Aeromonadales	*Aeromonadaceae*	*Aeromonas*	2
8.85	γ-proteobacteria	Alteromonadales	*Shewanellaceae*	*Shewanella*	3
1.85	γ-proteobacteria	Enterobacteriales	*Enterobacteriaceae*	*Enterobacter*	1
1.42	Bacteroidia	Bacteroidales	*Porphyromonadaceae*	*Parabacteroides*	2

**Figure 7:**
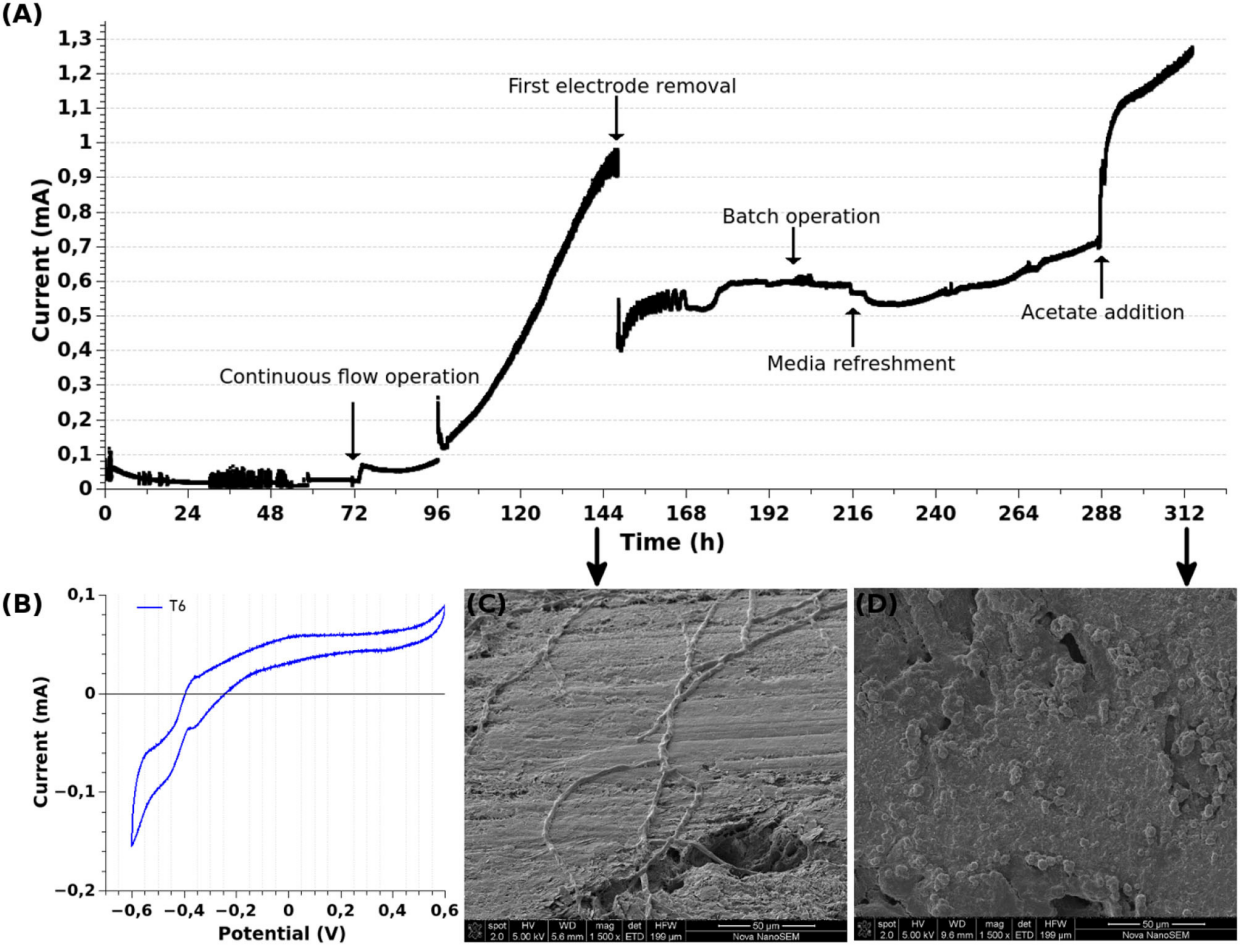
Electrochemical characterization of the selected community. A MEC with two graphite WEs was inoculated with the selected community. The WEs were at an applied potential of 480 mV (vs. Ag/AgCl). The MEC was operated at 30°C in anaerobiosis, other operational parameters were modified as specified in the figure itself. Continuous flow at a HRT = 24 h. **(A)** Chronoamperometry. **(B)** Cyclic voltammetry at 3 mV/s, T6 = 6 days of MEC operation (144 h), potential vs. Ag/AgCl. **(C,D)** Scanning electron microscopy of the first and second WEs, respectively, removed at the indicated time of MEC operation.

Next, in order to further analyze the biofilm development and behavior of the selected community, we prepared a new MEC with two graphite WEs. The WEs were at an applied potential of 480 mV (vs. Ag/AgCl). The current intensity was recorded and is shown in Figure 7A. A lag phase of ≈72 h was observed. Then, the cell operation was changed from batch to continuous mode with a hydraulic retention time of 24 h. Since then, the characteristic growth of electrogenic microorganisms could be observed.

At ~144 h of operation (T6), the curve began to flatten and hence the maximum current intensity was being reached. CV performed at this point (turnover conditions, Figure 7B) showed similar features to the one performed at T28 during the selection procedure: a catalytic wave at ≈−400 mV (vs. Ag/AgCl) and the presence of mediated and direct electron transfer mechanisms. Then, in order to evaluate the characteristics of the biofilm at this point of maximal current intensity, one of the electrodes was removed for SEM observation. As can be appreciated in Figure 7C, the biofilm was mostly composed of cables of bacteria running along the electrode surface. A detailed description of SEM inspection is given in the next section of the manuscript.

As expected, the removal of the electrode was evidenced in the chronoamperometry by the drop in the current intensity from ≈0.95 to 0.45 mA (Figure 7A; 144 h), which is due to the removal of half of the active surface. A plateau of current production was reached at ≈200 h. Considering that the acetate metabolic oxidation (Equation 1) produces 1 Coulomb per 1.295 μmol of acetate, at this point of maximal current production the rate of acetate consumption translated into current was 2.80 μmol h^-1^. Since the inflow of acetate to the cell was 250 μmol h^-1^, we could assume that acetate was in excess. Therefore, in order to gain insights into the biofilm behavior, the MEC operation was changed from continuous flow to batch mode again. In spite of the fact that acetate should have still been in excess after 24 h of batch operation (assuming a 100% of acetate conversion efficiency, the consumption of acetate after 24 h should have been 67.13 μmol), the current began to drop. This result could be due to different phenomena: acetate diffusion inside the biofilm was limited by the biofilm density and/or the acetate was being consumed by microorganisms which did not contribute to current production, limiting the acetate concentration accessible to the electrogenic bacteria.

(1) $$\begin{array}{l} {\text{CH}}_{3}{\text{COO}}^{-}+4{\text{H}}_{2}\text{O}\to 2{\text{HCO}}_{3}^{-}+9{\text{H}}^{+}+8{\text{e}}^{-} \end{array}$$

Therefore, aiming to understand the process, we refreshed the whole MEC medium (6 mmol acetate) and the MEC was left in batch mode. This procedure would provide more carbon and energy source and also remove possible toxic substances. As expected from the previous experiment, this change immediately induced a mild drop in the current intensity, which could be related to the removal of soluble electron shuttles. Then the current began to recover. The recovery was slower than the original current production (0.0032 and 0.0181 mA h^-1^, respectively), which was observed during continuous flow operation, probably indicating a diffusional limitation of acetate. Additionally, in a growing biofilm the acetate oxidation rate becomes limited by protons accumulation inside the biofilm (Torres et al., 2008). Therefore, as the biofilm gets thicker the respiration rate or current production gets slower unless proton transport out of the biofilm is favored (Lescano et al., 2018). The increase in current intensity in these conditions (MEC operated in batch mode with fresh medium) was longer than 24 h, suggesting that there was still acetate available for the electrogenic bacteria. However, at 287 h when the current intensity was still with an increasing tendency, we added more acetate (2 mmol). This addition induced an immediate and abrupt increase in current production (~55%). It also increased the rate of current production (0.0062 mA h^-1^). Even though this rate was faster than the one observed after the medium refreshment, it was slower than the one observed during the firsts days of operation under continuous flow. These results suggest that acetate might be limited to the electrogenic bacteria in the mature biofilm by diffusion and consumption by non-electrogenic members of the community. This hypothesis is further supported by SEM images of this electrode, which mostly show a highly packed and dense biofilm over the electrode surface (Figure 7D). In the next section of the manuscript are shown and discussed more images comparing the different stages of the biofilm development.

Altogether, these results confirm that the selected microorganisms are capable of growing by means of anode respiration. Additionally, the thickness or structure of the mature biofilm might be limiting the acetate diffusion to the inner layers of cells and the transport of protons outside the biofilm, thus limiting MEC performance. Moreover, there might be members of this community which consume acetate but do not contribute to current production.

### 3.5. The Selected Electrogenic Bacteria Colonized the Electrode by Means of Wiring Up Along the Electrode Surface

**Figure 8:**
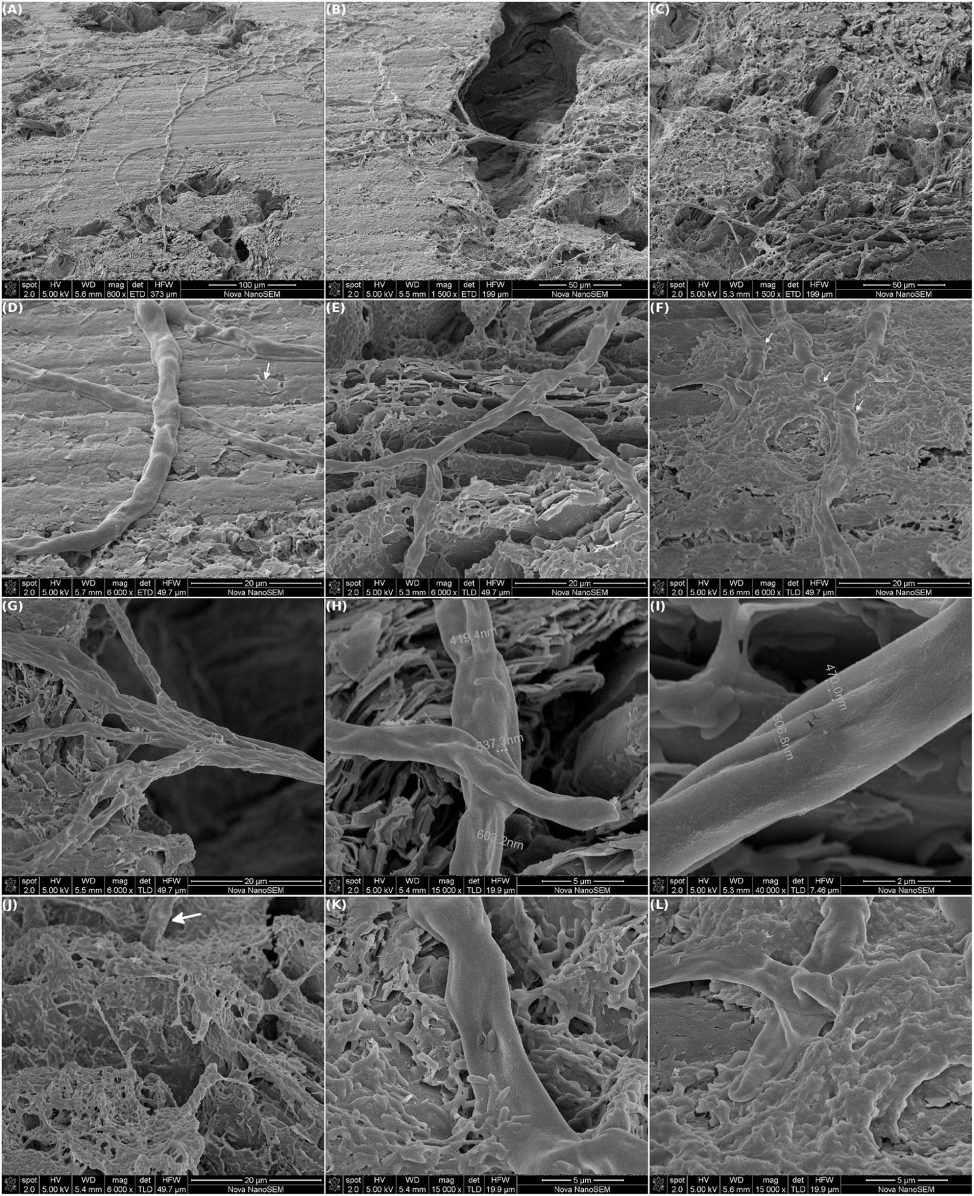
Scanning electron microscopy of one of the two working electrodes of a MEC inoculated with the selected community. The WEs (graphite) were polarized at +480 mV vs. Ag/AgCl. The MEC was operated at 30°C in anaerobiosis. At approx. 144 h of operation, one WE was removed and prepared for SEM. **(A–C)** Intertwined millimeter long cables of bacteria running along the WE surface. **(D–F)** Differences in the biofilm structure were observed over the electrode: zones of high density of cables and low density of isolated cells (**D**, white arrow), and zones with low density of cables and higher density of aggregated cells **(E)** and biofilm matrix (**F**, white arrows highlight some wrinkles in the cables). **(G–I)** The cables were composed of many single cables. **(J–L)** A non-cable type biofilm seemed to be growing over the cables.

Figure 8 shows SEM images of the first electrode that was removed from the MEC mentioned in the previous section, when the highest current intensity was being reached. Once again, an impressive net of cables of bacteria were observed running along the electrode surface. As described during the selection procedure, different areas of the electrode could be distinguished: areas with a high density of cables, a few isolated cells and plain graphite sheets; areas with a lower cable density and more aggregated cells and areas with a dense biofilm matrix (Figures 8D–F, respectively).

The cables observed in this occasion were wider than the ones seen after the selection procedure. Also, the current intensity at which the electrode was removed was higher, probably indicating that the growth of the cables was related to current production. As mentioned in that opportunity, each wide cable seemed to be the fusion of many individual cables (Figures 8G–I). The texture of the sheath that covered the cables was similar to the one described after the selection. Although, in this opportunity this sheath looked baggier (highlighted with white arrows in Figure 8F). Since the electrode was dehydrated and air-dried for SEM inspection the images shown might not reflect its actual structure during MEC operation, but they suggest that there might have been water beneath the sheath.

Additionally, a non-cable type biofilm was also growing immerse a dense matrix. In some cases, this biofilm seemed to be growing over the cables (Figures 8J–L). Such a microbial stratification has already been observed on an anode-respiring biofilm, where a *Geobacter* cluster was highly enriched in the innermost layers of the biofilm, which were in contact with the electrode, and the rest of bacteria were located in the outermost layers (Tejedor-Sanz et al., 2018).

**Figure 9:**
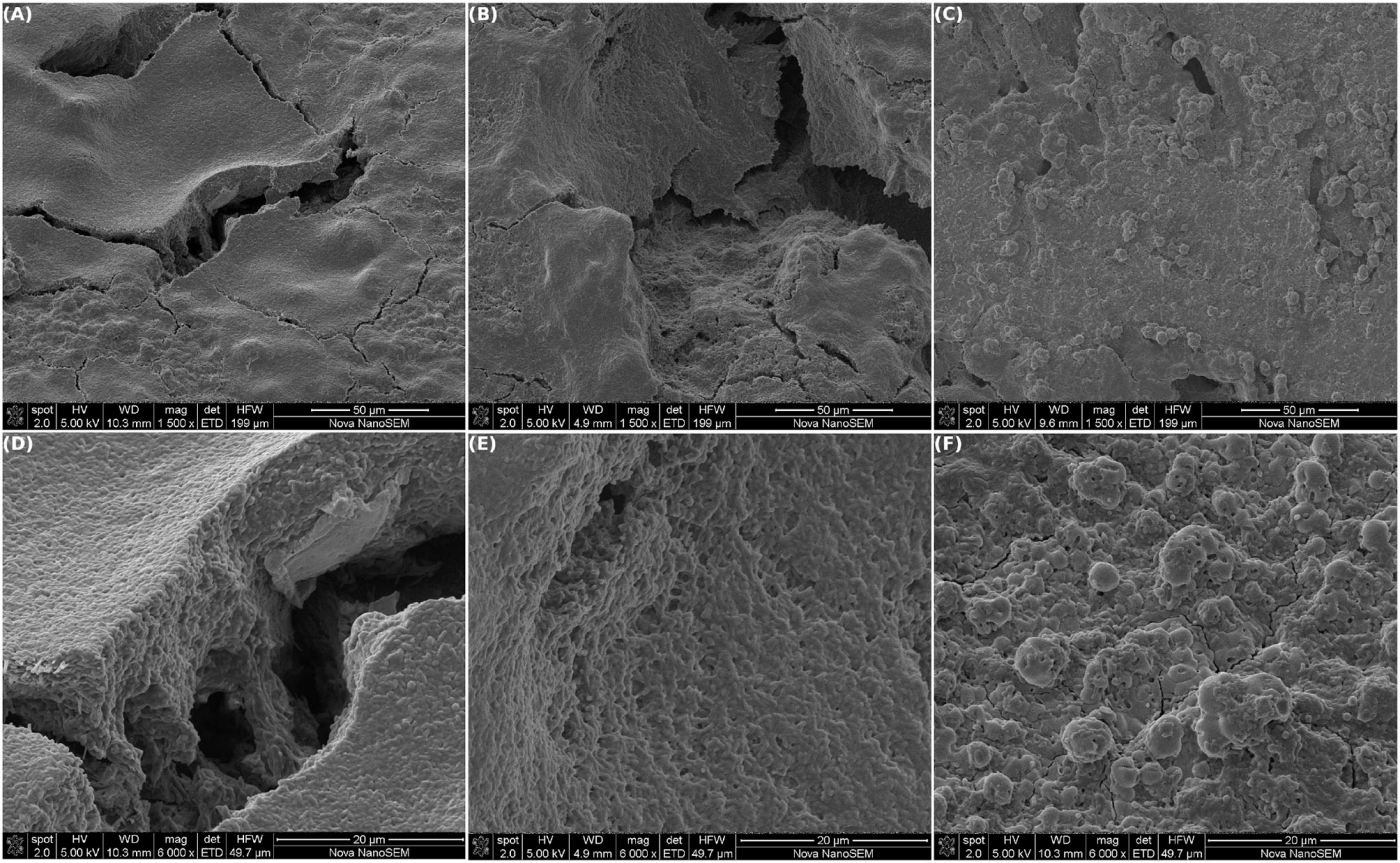
Scanning electron microscopy of one of the two working electrodes of a MEC inoculated with the selected community. The WEs (graphite) were polarized at +480 mV vs. Ag/AgCl. The MEC was operated at 30°C in anaerobiosis. At 312 h of operation the second WE was removed. Different areas of the graphite are shown at low **(A–C)** and high magnification **(D–F)**.

Figure 9 shows SEM images of the second electrode that was removed from the MEC mentioned in the previous section. In this case, the WE surface was completely covered with cells and the “typical” picture of dense electrogenic biofilms was observed (Kitayama et al., 2017; Lescano et al., 2018). However, even in this completely covered electrode surface, different biofilm structures could be distinguished. Figures 9A,B show areas of the electrode covered by a plain biofilm surrounded by an heterogeneous or uneven biofilm. No visible limit between these structures could be distinguished. Instead in Figure 9C a zone with a high density of columns is shown. Interestingly, higher magnification images of these zones revealed other engaging features. As can be appreciated in Figures 9D,E (higher magnification of Figures 9A,B, respectively), the biofilm included mostly bacilli that were evenly and densely packed. Additionally, in Figure 9D columns of cells arising from the electrode surface and supporting the packed biofilm can be clearly seen. This column-and-canopy-shaped biofilm was previously reported for a pure culture of *Pseudomonas aeruginosa* during spaceflight (Kim et al., 2013). In contrast, in Figure 9F (higher magnification of Figure 9C) the biofilm seemed to have mound-like structures with a great amount of EPS and different bacterial cell types. Additionally, in this case, imprints of cells that had left the biofilm can be easily appreciated.

Therefore, these results confirm that at least one of the members of the community develops a cable-type biofilm at the beginning of BES operation and this cable biofilm was related to the higher BES performance.

## 4. Discussion

Electrogenic microorganisms have caught microbiologist attention due to their unique capability of respiring solid extracellular compounds and electrodes. This aptitude has turned them into a promising biotechnological platform for environmental and industrial processes.

Numerous reports have already demonstrated that electrogenic microorganisms are widely distributed in natural ecosystems including marine, freshwater, sedimentary, and soil environments and can be enriched in bioelectrochemical systems (BES). Intriguingly, electrogenic communities rather than single electrogenic species are most often recovered from BES electrodes (Torres et al., 2009; Zhu et al., 2014; Li and Nealson, 2015). Nevertheless, in most of the cases, the specific interactions between the members of the community are not known and thus, the role of the “non-electrogenic” members are ignored.

The performance of the BES is commonly affected by the electrogenic members included in the different inoculum source (Ishii et al., 2017). Therefore, in this work, we developed a strategy to select electrogenic microorganisms from a polluted river sediment. The class-level community composition of the selected bacteria revealed a low microbial diversity. Only two taxonomic groups were highly abundant after the selection procedure: γ-Proteobacteria and Clostridia. A third taxonomic group, Bacteroidia, was present at a very low abundance. Among the highly abundant groups, a well-characterized electrogenic microorganism, *S. putrefaciens*, and four genera of potentially electrogenic bacteria, *Shewanella, Aeromonas, Enterobacter*, and *Clostridium*, were present. We consider these genera as “potentially electrogenic” since different species belonging to them have already been tested as pure cultures in BES and their electrogenic capabilities demonstrated (Kim et al., 1999; Park et al., 2001; Pham et al., 2003; Feng et al., 2014). However, in this work we could not determine (apart from *S. putrefaciens*) which particular specie was present in the community. On the other hand, considering the general role of the genera *Parabacteroides* (class Bacteroidia) regarding polysaccharides metabolism, it was suggested that this phylotype might contribute to polysaccharides degradation in the electrogenic community (Ishii et al., 2017). Even though these three taxonomic groups have been frequently reported in electrogenic communities, it is still not clear which type of interaction exists between the species that favors the selection of the community, whether they cooperate, compete or there is an equilibrium between the two.

Our results highlight the complexity and highly dynamic nature of a multi-species biofilm. The members of the community colonized the electrode and developed distinctive biofilm structures. An initial spatial segregation could be distinguished, where the surface of the electrode was mainly dominated by those bacteria capable of colonizing it by a cable-like biofilm forming strategy. To our knowledge, this is the first report of such a biofilm structure growing over a poised surface. Interestingly, this biofilm was associated with the higher current production rate. As the biomass increased, a layered structure with a patchy pattern of different types of biofilms (probably involving different bacterial species) was observed. This type of multi-species biofilm structure is believed to be the result of an initial weak interdependence of the interacting species, followed by competition as the biofilm grows and finally exploitation of one specie over another, as stated by Liu et al. (2016). This observation is in accordance with the chronoamperometry results, where the maximal current intensity was reached with the cable-type biofilm, but as the biofilm grew current production was limited by acetate diffusion and consumption by “non-electrogenic” members of the community. Thus, in spite of the fact that it is established that multi-species biofilms are important for several technological applications, we need to further investigate the microbial interactions during BES operation in order to understand the complete biological processes that are taking place and which will enable the optimization of BES performance.

It is important to mention that Reimers et al. (2017) also observed multi-cellular mm-long filaments of bacteria attached to the anode of a benthic microbial fuel cell, which had been operated in an estuarine environment for over a year. These filaments were identified as “cable-bacteria,” belonging to the *Desulfobulbaceae* family. Two candidate genera of “cable-bacteria” were later proposed: *Candidatus Electrothrix* and *Candidatus Electronema* (Trojan et al., 2016). None of these genera were identified in the community selected here. Additionally, several distinct features were observed between the cables found in this work and those formed by “cable-bacteria.”

As described by Reimers et al. (2017), “cable-bacteria” individual filaments were attached to the anode by single terminus cells with networks of pilus-like nano-filaments radiating out from these cells. It was suggested that the nano-filaments might have been produced by the genera *Desulfuromonas*, which formed a consortium with the “cable-bacteria.” In our work, the mm-long filaments were observed over the electrode surface and had several points of intimate contact with the electrode, including the terminal cell and the extremes of the incredible straight bridges that were attached to the edges of the graphite macro-pores. No nano-filaments were observed, accordingly the order Desulfuromonadales was not identified in the community (the pilA gene responsible for the proper assembly of nano-filaments appears to be restricted to this order, Holmes et al., 2016). Furthermore, “cable-bacteria” build linear, not branched filaments as the result of cell division distributed along the filament (Geerlings et al., 2021). In our work, branched filaments could be observed and the elongation of the filaments might be mediated also by the connection of individual cells by nanotubes.

In addition, a characteristic feature of “cable-bacteria” is the ridge topography of the cable that is usually observed by SEM (Pfeffer et al., 2012; Risgaard-Petersen et al., 2015; Trojan et al., 2016). These ridges are parallel periplasmic fibers, which are continuous across cell-to-cell junctions and are the electron conducting structure in “cable-bacteria” that enable long-distance electron transport (Cornelissen et al., 2018; Meysman et al., 2019). Ridges were not observed in our preparations and cell-cell boundaries were hardly distinguishable. Our electrochemical results suggest that outer-membrane c-type cytochromes (redox potential compatible with Mtr/Omc) and, probably self-produced soluble electron-shuttles are the electron transfer mechanisms operating in the biofilm.

Several lines of evidence obtained in this work point out at *Shewanella* as the cable-forming bacteria of the community, capable of transferring electrons to the electrode. To begin with, the cyclic voltammograms obtained during the selection procedure and in the following experiments were highly similar to CVs from pure cultures of *Shewanella*, particularly *S. putrefaciens* (Carmona-Martínez et al., 2013), and were distinct from other cultures such as those of “cable-bacteria” (Meysman et al., 2019). Additionally, the observed response to a higher applied potential was also in accordance with *Shewanella* spp. (Carmona-Martínez et al., 2013; Matsuda et al., 2013; Hirose et al., 2018). Interestingly, this increase in current production as a function of the applied potential is related to a change in the metabolic activity of the cells (Hirose et al., 2018).

Additionally, it was previously demonstrated that *Shewanella oneidensis MR-1* can have a filamentous phenotype as a response to changes in culture conditions (Abboud et al., 2005). However, the meaning of the filamentous phenotype could not be elucidated. Also, *Shewanella* can develop different biofilm structures in response to the BES operation mode (batch vs. electrolyte-flow conditions) and the applied potential (Kitayama et al., 2017). Nonetheless, none of these different biofilm structures were a cable-type biofilm. In our case, since the cable biofilm was observed both during the selection procedure (BES operated in batch) and in the following experiments (BES operated in continuous flow), we believe that this structure was not related to the BES operation mode. Instead, this biofilm could have been induced by the applied potential, which was higher than the usually tested. We are currently working on the isolation of the members of the community in order to address these hypotheses.

On the other hand, Pirbadian et al. (2014) demonstrated that the so called *Shewanella* “nanowires” are outer membrane and periplasmic extensions of the extracellular electron transport components. Under anaerobic conditions, *Shewanella* produces “nanowires” through a mechanism that involves the production of chained membrane vesicles (blebbing) that are finally converted into a filament. It was suggested that these filaments mediated extracellular electron transfer and that they could also increase the chance of cells to encounter solid electron acceptors by increasing the surface area-to-volume ratio of the cells. The growing of “nanowires” from one cell to connect another individual cell was impressively documented. Since *Shewanella* “nanowires” are distinct from the *Geobacter* conductive filaments (Wang et al., 2019), which are also referred as “nanowires,” Lovley and Malvankar (2015) proposed the term *nanopods* instead of “nanowires” to distinguish the membrane extensions of *Shewanella* from the *Geobacter* filaments. However, we prefer the term *nanotubes*, which were previously described as tubular extensions bridging neighboring cells. These structures serve as a route for the exchange of intra-cellular molecules and were reported to occur even between different bacterial species (Dubey and Ben-Yehuda, 2011).

Therefore, considering the detailed examination of scanning electron micrographs presented here (e.g., individual cell blebbing, cable blebbing, nanotubes connecting cells, and smooth sheath covering the cables), we hypothesize that the cables of bacteria are the result of the fusion of the cells by means of nanotubes, which are composed of an outer membrane that finally shapes the cable sheath. Thus, the blebbings or vesiculation during biofilm growth over the electrode could be the initial step of outer membrane extensions that may finally end up in nanotubes that link the periplasmic space of the cells. Accordingly, Remis et al. (2013) observed the up-regulation of outer membrane-derived extracellular membrane extensions (in the form of vesicle chains, periodically restricted tubes and round membrane tubes) in biofilms of another Gram-negative bacteria (*Myxococcus xanthus*).

In addition, Reardon et al. (2010) clearly demonstrated the role of *Shewanella* outer-membrane cytochromes MtrC and OmcA in the biomineralization of ferrihydrite. After the reduction of ferrihydrite, crystal phases grew on the cell surface, being the predominant phases ferrous phosphates, including vivianite [Fe_3_(PO_4_)_2_.8H_2_O]. This is in accordance with our observation of crystals specifically deposited over the surface of the cables of bacteria, which were mainly composed of Fe and P. Vivianite crystals were identified by X-ray diffraction and SEM of the precipitates obtained after culturing the selected community in the presence of Fe-citrate (unpublished data). It is important to note that the crystals were only observed in the cables from the electrode employed during the selection procedure since the inoculum included the Fe-citrate from the enrichment medium (Fe-citrate was absent in the subsequent experiments). Furthermore, in this electrode Fe was also detected in the EPS, though in this case no precipitates were distinguished. Interestingly, it was demonstrated that EPS operate in Fe homeostasis by sorbing Fe, which can later be released and taken up by the cells when required (Jittawuttipoka et al., 2013).

Finally, the fact that the cable-type biofilm dominated the first stages of the biofilm development suggests that this structure may represent an advantageous adaptative response of the implied cells. This response allowed the cells to rapidly colonize a big surface area, reaching and spanning over the whole solid electron acceptor. Noteworthy, a bacterial behavior named electrokinesis was first described for different *Shewanella* strains (Harris et al., 2009). This behavior was characterized by an increase in cell swimming speeds and lengthened paths of motion, occurring near a redox active mineral surface or a polarized electrode. Electrokinesis required functional extra-cellular electron transport, such as the outer-membrane cytochromes (Mtr) that were detected in this work early during the selection procedure. Thus, it would be interesting to address whether this behavior is implied in the building of the cable biofilm. If so, it could be speculated that the cable biofilm could also be developed in the presence of other solid electron acceptors, such as mineral surfaces, in natural environments.

Ultimately, it seems that *cable bacteria or bacteria capable of forming cables* are diverse in nature and they may have different electrical properties. This differential features could be harnessed not only to improve bioelectrochemical systems applications but also to contribute to innovative research and development nanomaterials areas, like bioelectronics.

## 5. Future Research

The results presented in this work contribute to the growing body of knowledge about electrogenic bacteria by showing an amazing way of colonizing a polarized surface and employing it for cellular respiration. Many questions for future research arise from this work, such as: which bacteria are capable of cable-type biofilm growing? Are the cables composed of a single specie of bacterium? Are the cables only the result of the electrode polarization or the interaction between the different genera also induced their development? Do the cells in the cable cooperate for electron transport or also share nutrients through nanotubes? Is the whole sheath that cover the cables a conductive outer-membrane or does it act as an insulator? How do the cables transfer electrons to the electrode?

## Data Availability Statement

The raw data supporting the conclusions of this article will be made available by the authors, without undue reservation.

## Author Contributions

MP conducted the research, performed experiments, followed-up analyses, recognized the discovery, and prepared the manuscript. ML performed experiments, followed-up analyses, and revised the manuscript. NP collected and prepared field samples. GC discussed experiments and results, critically reviewed the manuscript, and was responsible for part of the funding acquisition. All authors contributed to the article and approved the submitted version.

## Conflict of Interest

The authors declare that the research was conducted in the absence of any commercial or financial relationships that could be construed as a potential conflict of interest.

## Publisher's Note

All claims expressed in this article are solely those of the authors and do not necessarily represent those of their affiliated organizations, or those of the publisher, the editors and the reviewers. Any product that may be evaluated in this article, or claim that may be made by its manufacturer, is not guaranteed or endorsed by the publisher.
