# Supplementary material for: Wiring Up Along Electrodes for Biofilm Formation
Source: Front Microbiol. 2021 Aug 30;12:726251. doi: 10.3389/fmicb.2021.726251 (PMC8435748; doi:10.3389/fmicb.2021.726251)
Supplement: Supplementary file 1 [file Data_Sheet_1.pdf]

Supplementary Material

1 SUPPLEMENTARY TABLES AND FIGURES

1.1 Figures

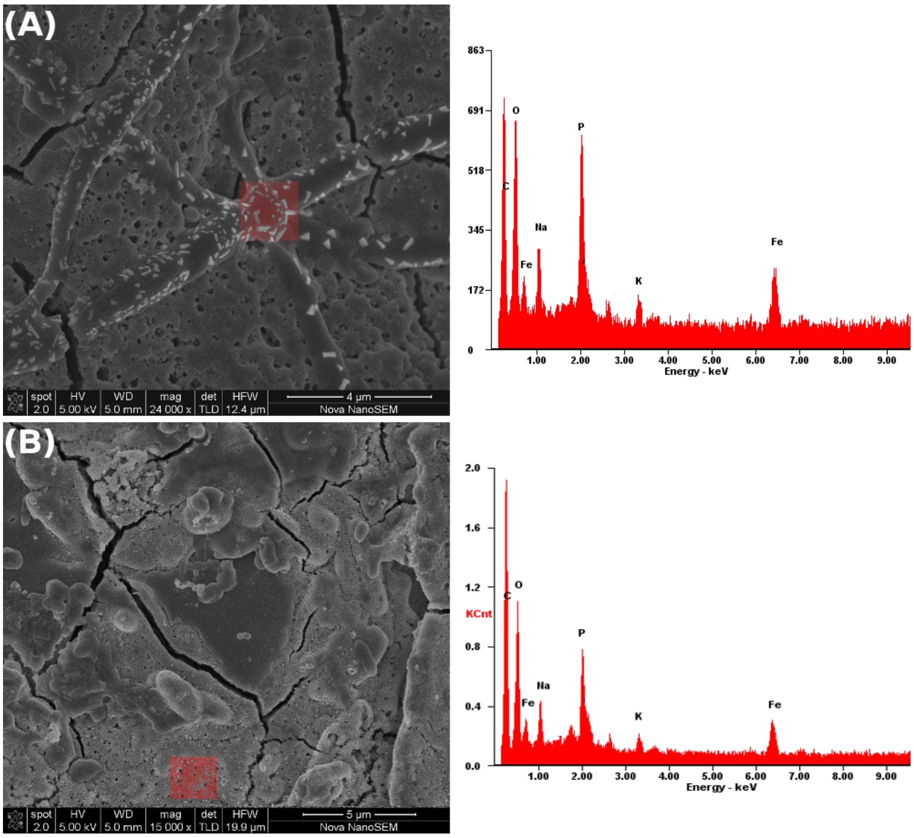

Figure S1. Energy-dispersive X-ray spectroscopy (EDS) of different areas of the MEC-a working electrode. The red block over the image indicates the area where EDS was performed. **(A)** Crystals over cables of cells. **(B)** Deposit over the WE.

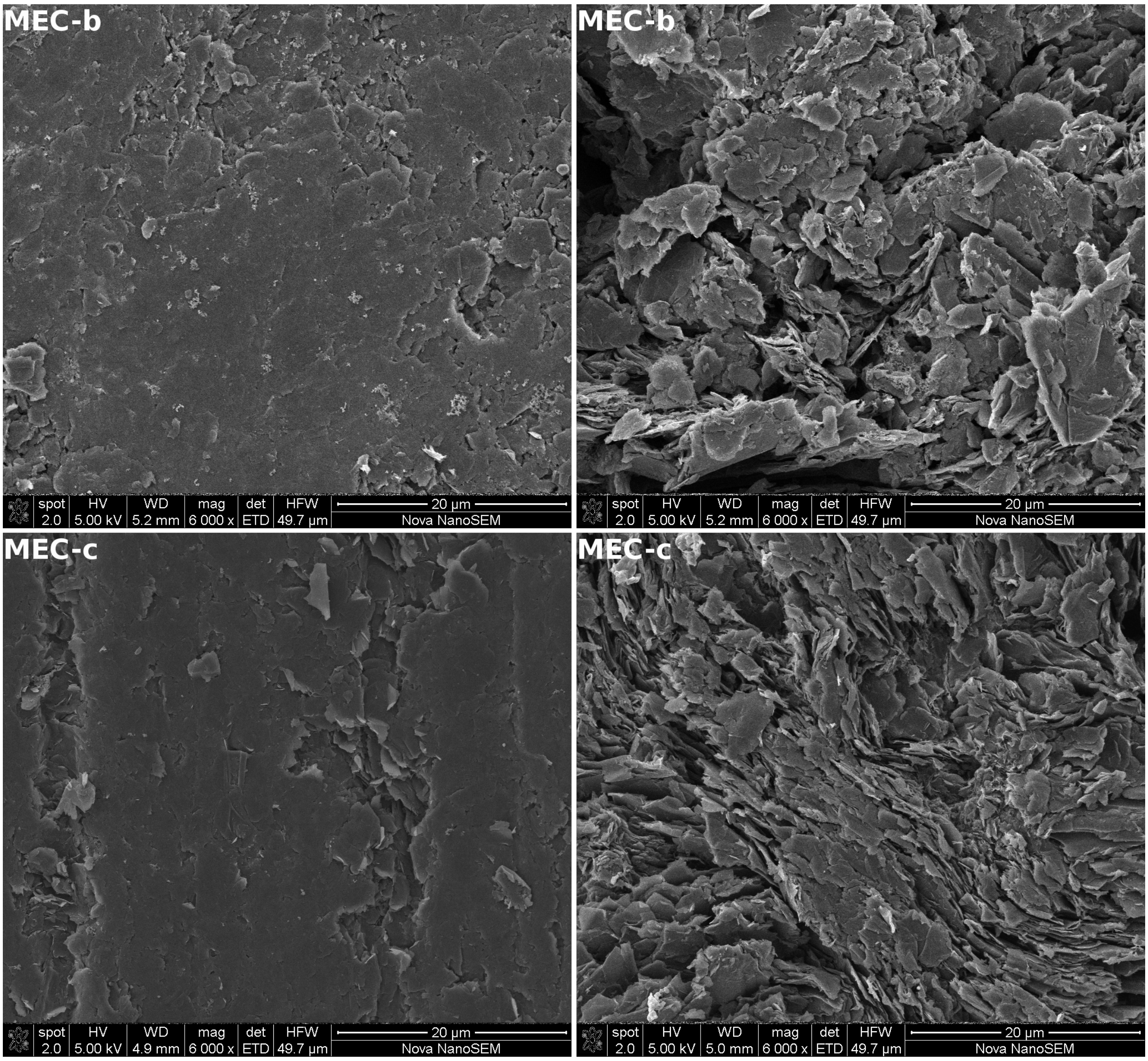

Figure S2. Scanning electron microscopy of MEC-b and MEC-c working electrodes (WE). MEC-b was inoculated with the Fe-reducing microorganisms enriched from the river sediment and the electric circuit was left open. MEC-c was inoculated with the control vial and the WE (graphite) was initially polarized at +240 mV vs. Ag/AgCl. The MECs were operated at 30° C in anaerobiosis. At the end of the experiment (41 days) the WEs were removed and observed by SEM: no significant bacteria attachment was observed in any of these WE.
